# Three myths about risk thresholds for prediction models

**DOI:** 10.1186/s12916-019-1425-3

**Published:** 2019-10-25

**Authors:** Laure Wynants, Maarten van Smeden, David J. McLernon, Dirk Timmerman, Ewout W. Steyerberg, Ben Van Calster, on behalf of the Topic Group ‘Evaluating diagnostic tests and prediction models’ of the STRATOS initiative

**Affiliations:** 1 KU Leuven Department of Development and Regeneration, Leuven, Belgium; 2 Department of Epidemiology, CAPHRI Care and Public Health Research Institute, Maastricht University, Maastricht, The Netherlands; 3 Department of Clinical Epidemiology, Leiden University Medical Center, Leiden, The Netherlands; 4 Department of Biomedical Data Sciences, Leiden University Medical Center, Leiden, The Netherlands; 5 Medical Statistics Team, Institute of Applied Health Sciences, School of Medicine, Medical Sciences and Nutrition, University of Aberdeen, Aberdeen, UK; 6 Department of Obstetrics and Gynecology, University Hospitals Leuven, Leuven, Belgium

**Keywords:** Clinical risk prediction model, Threshold, Decision support techniques, Risk, Data science, Diagnosis, Prognosis

## Abstract

**Background:**

Clinical prediction models are useful in estimating a patient’s risk of having a certain disease or experiencing an event in the future based on their current characteristics. Defining an appropriate risk threshold to recommend intervention is a key challenge in bringing a risk prediction model to clinical application; such risk thresholds are often defined in an ad hoc way. This is problematic because tacitly assumed costs of false positive and false negative classifications may not be clinically sensible. For example, when choosing the risk threshold that maximizes the proportion of patients correctly classified, false positives and false negatives are assumed equally costly. Furthermore, small to moderate sample sizes may lead to unstable optimal thresholds, which requires a particularly cautious interpretation of results.

**Main text:**

We discuss how three common myths about risk thresholds often lead to inappropriate risk stratification of patients. First, we point out the contexts of counseling and shared decision-making in which a continuous risk estimate is more useful than risk stratification. Second, we argue that threshold selection should reflect the consequences of the decisions made following risk stratification. Third, we emphasize that there is usually no universally optimal threshold but rather that a plausible risk threshold depends on the clinical context. Consequently, we recommend to present results for multiple risk thresholds when developing or validating a prediction model.

**Conclusion:**

Bearing in mind these three considerations can avoid inappropriate allocation (and non-allocation) of interventions. Using discriminating and well-calibrated models will generate better clinical outcomes if context-dependent thresholds are used.

## Background

Risk prediction models yield predictions for patients at risk of having a certain disease or experiencing a certain health event in the future. They are typically constructed as regression models or machine learning algorithms that have multiple predictors as inputs and a continuous risk estimate between 0 and 1 as output [1, 2]. The calculated risk for a specific individual supports healthcare professionals and patients in making decisions about therapeutic interventions, further diagnostic testing, or monitoring strategies. The underlying goal in many applications is risk stratification, such that high-risk patients can receive optimal care while preventing overtreatment in low-risk patients. This triggers the question: how should the risk threshold to differentiate between risk groups be determined?

The popular appeal of simplistic methods to analyze data has affected the published scientific literature [3–5]. One well-known example is ‘dichotomania’, the practice of imposing cut-offs on continuous variables (e.g., replacing the age in years by a categorical variable dividing patients into two groups, < 50 and ≥ 50 years old). Many have illustrated that artificially categorizing data can be detrimental for an analysis [1, 6, 7]. The recommended approach is therefore to maintain continuous risk factors continuous in the analysis. In the context of risk prediction, the categorization of a predictor leads to a premature decision about meaningful and clinically useable risk groups, and is thus a waste of information. If risk groups are desired, these should be defined based on a model’s predicted output instead of its inputs.

Regrettably, thresholds to divide patients into groups of predicted risk are often defined in an ad hoc way, lacking clinical or theoretical foundation. For example, thresholds are often derived by optimizing a purely statistical criterion (e.g., the Youden index or the number of correct classifications) for a specific dataset, without realizing that this threshold may be inappropriate in clinical practice or that a different threshold would be obtained if another dataset from the same population were used (for some published examples, see [8–12]). It is also not uncommon for researchers to present the sensitivity of a risk model without specifying the threshold that was applied. The international STRengthening Analytical Thinking for Observational Studies (STRATOS) initiative (http://stratos-initiative.org) aims to provide accessible and accurate guidance documents for relevant topics in the design and analysis of observational studies. In what follows, we visit three common myths about risk thresholds and attempt to explain the strengths and weaknesses of alternative ways to determine thresholds in a general and critical way. The R code and data to replicate this analysis are available as Additional file 1 and Additional file 2.

### Myth 1: risk groups are more useful than continuous risk predictions – no, continuous predictions allow for more refined decision-making at an individual level

Any classification of the predicted risk implies a loss of information because everyone within a class is treated as if they have the same risk. Individuals whose risks estimates are similar but are on either side of the risk threshold are assigned different levels of risk, and potentially different treatments. In contrast, a calibrated continuous risk on a scale from 0 to 1 allows for more refined decision-making. A predicted risk of cancer of 30% means that, among 100 women with such a predicted risk, you would expect to find 30 malignancies. This extra information may, in practice, lead to different patient management than when the patient had been labeled as ‘low-risk’ (as 30% is below the threshold).

A crude classification in broad risk categories is undesirable in many cases, especially when discrimination is poor with a large overlap in predicted risks for cases and non-cases (low area under the receiver operating characteristic curve (AUC, Table 1), or when the clinical context calls for shared decision-making. In these cases, a calibrated continuous risk estimate is more informative and allows patients to set their own thresholds. For example, a personalized risk estimate of the probability of pregnancy is of great value to inform and counsel subfertile couples, despite moderate discrimination between couples that do and do not conceive [13].

**Table 1:** Common terms

AUC	Area under the curve, in this case the receiver operating characteristic curve. A measure of discrimination. For prediction models based on logistic regression, this corresponds to the probability that a randomly selected diseased patient had a higher risk prediction than a randomly selected patient who does not have the disease.
Calibration	Correspondence between predicted and observed risks usually assessed in calibration plots or by calibration intercepts and slopes.
Sensitivity	The proportion of true positives in truly diseased patients.
Specificity	The proportion of true negatives in truly non-diseased patients.
Positive predictive value	The proportion of true positives in patients classified as positive.
Negative predictive value	The proportion of true negatives in patients classified as negative.
Decision curve analysis	A method to evaluate classifications for a range of possible thresholds, reflecting different costs of false positives and benefits of true positives.
Net reclassification improvement	Net reclassification improvement, reflecting reclassifications in the right direction when making decisions based on one prediction model compared to another.
STRATOS	STRengthening Analytical Thinking for Observational Studies

In other cases, guidelines recommend interventions based on risk thresholds. Here, communicating an individuals’ personal risk estimate and comparing it to the proposed threshold may also improve counseling and allow a discussion of diagnostic and therapeutic options. In contrast, other situations require efficient triage or immediate action (e.g., in emergency medicine) and leave little room for deliberation. Here, risk groups coupled to recommendations regarding treatment or management facilitate decision-making.

### Myth 2: your statistician can calculate the optimal threshold directly from the data – no, a good threshold reflects the clinical context

**Fig. 1:**
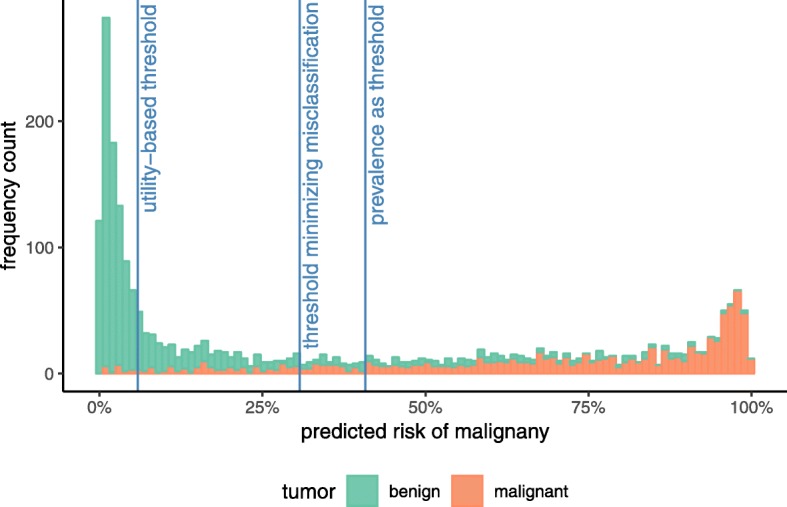
Frequencies of predicted risks of malignancy and three possible risk thresholds

In using a risk model as a classification rule and a decision aid, one intervenes (e.g., proceeds with the diagnostic workup or treats) if an individual has a predicted risk equal to or exceeding a certain risk threshold, ‘t’, and one does not intervene if the risk falls below ‘t’. Consider the ADNEX model that predicts an individual’s risk of having ovarian cancer (Table 2). Patients with a predicted probability of cancer higher than the prevalence in the dataset (0.41) may be classified as high-risk, whereas patients with a lower predicted probability may be classified as low-risk (Fig. 1) [15]. The holy grail of thresholds is to define risk groups without misclassification – a low-risk group in which cancer does not occur and a high-risk group that surely benefits from further testing and/or treatment. In reality, false negative (below the threshold, but diseased) and false positive (above the threshold, but healthy) classifications are unavoidable. The threshold that minimizes misclassification is the threshold where the sum of the number of false positive and false negative results is lowest. As can be seen in Fig. 1, the bins on the left side of that threshold are partly red and bins on the right side are partly blue, even though the ADNEX model has exceptionally good discrimination compared to most other published risk models.

**Table 2:** Example of a risk model: the ADNEX model

ADNEX is a model to preoperatively characterize ovarian cancer by calculating the risks of benign tumors and four classes of malignant tumors. It was constructed using multinomial logistic regression and validated with more recent data and in other centers [14]. Its predictors are age, CA125 levels, the tumor diameter, the proportion of solid tissue in the tumor, the number of locules, the number of papillary projections, the presence of acoustic shadows, the presence of ascites, and whether the patient was seen at a tertiary oncology center.

An alternative is to choose a threshold based on how each possible outcome is valued – a true positive, false positive, true negative and false negative each have their own value or ‘utility’. The costs of false negative (C_FN_) and false positive (C_FP_) classifications can be expressed in terms of mortality and morbidity, or even in arbitrary units combining multiple costs and patients’ personal values (Table 3) [26]. In the ADNEX example, we will consider the percentage of patients with severe morbidity and mortality (Table 4) [27–29]. The cost of a false negative may be estimated to be 95, reflecting the probability of severe morbidity and mortality among false negatives, caused by the delay in diagnosis and by treatment by general surgeons or gynecologists rather than referral to a gynecological oncology unit. To a false positive, we attribute a cost of 5, reflecting the complication risk when a benign tumor is surgically removed for staging (e.g., injury to hollow viscus, deep vein thrombosis, pulmonary embolism, wound breakdown, bowel obstruction, myocardial infarction). True positives have a cost (C_TP_) too, since some patients die or suffer severe morbidity despite early detection. In addition, laparotomy and chemotherapy treatment may cause morbidity. We estimate the percentage with severe morbidity and mortality among true positives to be 15. The cost of a true negative (C_TN_) is the cost of the ultrasound investigation to compute the ADNEX risk, which is set to 0 because ultrasound is considered a very safe imaging technique.

**Table 3:** Health-economic perspectives and clinical judgment in prediction modeling

Risk thresholds ideally reflect the clinical context by balancing the benefits of correct decisions against the costs of incorrect decisions. Health economists often prefer to value outcomes of decisions in terms of quality-adjusted life-years, which combine mortality and quality of life in a single measure. Utility values (like quality of life) can be elicited using various formal methods [16], and physicians’ judgements tend to differ from patients’ views, which ultimately matter more [17–19]. Thus, quantifying costs and benefits (or ‘utilities’) should be done carefully [20].	
In addition, health policy frequently involves a trade-off between monetary costs and health outcomes. To reach a societal optimum, monetary costs need to be calculated from the societal perspective (rather than the perspective of the healthcare provider or the individual patient), by including, for example, lost productivity due to time off work [21]. In theory, a risk threshold can be determined to minimize a composite of clinical outcomes and monetary cost, but this would require expression of the willingness-to-pay [16, 21, 22]. The details of costs, utilities and loss functions to optimize the threshold in health economics are beyond the scope of the current paper.	
Besides data on costs and benefits [23], the results of validation studies describing the predictive performance of risk models are also instrumental to optimize the risk threshold in a health economic analysis. Briefly, if a risk prediction model is perfectly calibrated, the threshold is a function of the costs or utilities alone [24]. If not perfectly calibrated, the prevalence in the population and the sensitivity and specificity of the model at each potential threshold also play a role [16, 21]. Either way, measures of calibration and discrimination tend to be over-optimistic when calculated on the model development data, and often vary with the disease prevalence. Thus, it is crucial to obtain reliable estimates of predictive performance, preferably from external validation studies in independent data from the target population [1, 2]. It would be a premature decision to determine the risk threshold before the predictive performance is thoroughly investigated.	
While predictive performance is one input determining the optimal threshold, reliable data on costs or utilities are often not available in the process of validating a risk prediction model. Fortunately, the prediction modeler does not have to find the most optimal threshold from a health economic perspective to evaluate a model’s predictive performance. At the stage of model validation, it is often sufficient to consider a broad range of reasonable risk thresholds. This range can be set by asking for sensible upper and lower bounds on the maximum number of false positives one would tolerate to find one true positive [23]. For example, if a detected ovarian cancer is worth 16 unnecessary surgical interventions, an appropriate risk threshold for surgery would be 1/(1 + 16) = 6%. A risk-averse person would perhaps tolerate more unnecessary surgical interventions and motivate a lower bound on the risk threshold of 1%. In a clinical context, and in particular with severe illness, the upper bound on the risk threshold usually does not exceed 50% – an undetected case is generally considered more harmful than a false positive case. (For another example of setting a range of reasonable thresholds, see [23].) This chosen range of reasonable thresholds can be used to show how the model performs with different thresholds.	
It is only after a risk model is validated that a health economic analysis could optimize the risk threshold, based on the model’s demonstrated predictive performance, its positioning in the care pathway (e.g., in a sequence of tests [25]), the available healthcare resources, the disease prevalence, and the harms and benefits of decisions. Developing a model, validating the predictive performance, and determining a risk threshold are separate and demanding tasks.

**Table 4:** Costs of outcomes when making a decision based on a risk threshold

	Diseased	Not diseased
Intervene (predicted risk ≥t)	C_TP_ = 15 The cost of detected/treated disease, e.g., risk of death or severe morbidity despite detection, plus the cost of intervening	C_FP_ = 5 The cost of an unneeded intervention, e.g., invasiveness of testing, complication risks of treatment
Do not intervene (predicted risk <t)	C_FN_ = 95 The cost of an undetected disease, i.e., the risk of death or severe morbidity	C_TN_ = 0 The cost of applying the risk model

The risk threshold can be chosen to minimize the expected total costs [24, 30]. For a calibrated risk model, it can be determined as:

1 $$ t=\frac{C_{FP}-{C}_{TN}}{C_{FP}+{C}_{FN}-{C}_{TP}-{C}_{TN}}. $$

When the cost of a true negative is set to zero, this equals to:

2 $$ \frac{C_{FP}}{C_{FP}+{B}_{TP}}, $$

Here, the *benefit* (B_TP_) of a true positive classification, or intervening when needed, is the difference between the cost of a false negative and the cost of a true positive. In our example, this is 95–15 = 80. If one accepts the cost estimates given in Table 4, considering the harm of a false positive cancer diagnosis (C_FP_ = 5) as 16 times smaller than the benefit of a true positive cancer diagnosis (B_TP_ = 80), the threshold for the ADNEX model would be 5/(5 + 80) = 0.06, or 6% (Fig. 1). Alternatively, more complex, model-based analyses could be conducted to find (sub)population- and intervention-specific risk thresholds at which the benefits of intervening outweigh the costs and harms, taking into account a multitude of benefits and costs associated with intervening, as well as stakeholders’ (e.g., patients) preferences and values (for some examples, see [22, 31, 32]).

A purely data-driven rule to define the risk threshold makes (often implicit) assumptions on the costs. For example, minimizing the number of misclassifications for the dataset at hand assumes equal costs for a false positive and a false negative classification, and no costs for correct classifications [24, 30]; this is rarely appropriate. Moreover, a data-driven risk threshold is subject to sampling variability. With a different sample, a different threshold could be optimal. Thus, in a new sample, diagnostic accuracy is often lower, especially when datasets are small. Analyses should take this uncertainty into account [33, 34].

The appropriate threshold clearly depends on the clinical context. To decide on invasive surgery would typically require a higher risk threshold than deciding to send the patient for magnetic resonance imaging. In healthcare systems with long waiting lists for specialized care, false positives may be attributed higher costs than what is given here, as they delay treatment for patients who do need it. In addition, reliable data on cost and benefit estimates are rarely available, and if they are, they may not be transportable in time and space. The best risk threshold is therefore not directly derivable from the dataset used to develop or validate a risk model.

### Myth 3: the threshold is part of the model – no, a model can be validated for multiple risk thresholds

A risk prediction model can be used in multiple clinical contexts. In practice, reliable data on all costs involved are often difficult to collect and may vary between healthcare systems. Thus, the calculation of a universal risk threshold for decision-making is often impossible. Even a widely agreed-upon threshold may be subject to change and debate. For example, in 2013, commonly accepted thresholds for primary prevention of cardiovascular disease were lowered by the American Heart Association/American College of Cardiology guidelines, while the subsequent U.S. Preventive Services Task Force guideline raised the threshold [35, 36]. The current threshold is still too low according to detailed recent analyses of harms and benefits [31, 35, 36]. This has implications for performance evaluation of prediction models, as it would be undesirable for performance measures to reflect an arbitrarily chosen risk threshold. The statistical evaluation of predictions can be done without having to choose risk thresholds, by means of the AUC and measures of calibration that assess the correspondence between predicted and observed risks (Table 1) [1, 2].

Measures of classification, in contrast, do require risk thresholds. Researchers often present a model’s sensitivity, specificity, positive predictive value or negative predictive value (Table 1). These measures are all derived from a cross-tabulation of classifications with the true disease status after applying a risk threshold. Although these statistics have easy clinical interpretations, they have several limitations [25]. One limitation is that their values depend heavily on the chosen risk threshold. It is crucial to realize that there is no such thing as ‘the’ sensitivity of a risk model. Sensitivity and negative predictive value increase to a maximum of one as the threshold is lowered, while specificity and positive predictive value rise to a maximum of one as the threshold is increased (Table 5). Thus, when developing and validating a risk model, a reasonable alternative is to consider a range of acceptable risk thresholds, reflecting different assumed costs (Table 3) [23, 37]. In Table 5, we focus on thresholds up to 50%, reflecting that the benefit of a true positive outweighs the harm of a false positive.

**Table 5:** Classification statistics for a selection of thresholds

Threshold	Sensitivity (95% CI)	Specificity (95% CI)	Positive predictive value (95% CI)	Negative predictive value (95% CI)
0.1%	1.00 (1.00–1.00)	0.00 (0.00–0.01)	0.41 (0.39–0.43)	1.00 (0.40–1.00)
6% (Utility-based for costs in Table 4)	0.98 (0.97–0.99)	0.61 (0.59–0.64)	0.64 (0.61–0.66)	0.98 (0.96–0.98)
10%	0.97 (0.95–0.98)	0.70 (0.67–0.72)	0.69 (0.66–0.71)	0.97 (0.96–0.98)
20%	0.93 (0.91–0.94)	0.80 (0.78–0.82)	0.76 (0.73–0.78)	0.94 (0.92–0.95)
31% (minimize misclassification)	0.88 (0.86–0.90)	0.85 (0.83–0.87)	0.80 (0.78–0.83)	0.91 (0.89–0.93)
41% (prevalence)	0.83 (0.80–0.85)	0.88 (0.86–0.90)	0.83 (0.80–0.85)	0.88 (0.86–0.90)
50%	0.76 (0.74–0.79)	0.90 (0.89–0.92)	0.85 (0.82–0.87)	0.85 (0.83–0.86)
99.9%	0.00 (0.00–0.01)	1.00 (1.00–1.00)	1.00 (0.02–1.00)	0.59 (0.57–0.61)

Additionally, a decision curve analysis can be presented to evaluate a model’s clinical utility for decision-making. A decision curve is a plot of net benefit for a range of relevant risk thresholds, where net benefit is proportional to the number of true positives minus the number of false positives multiplied by $\frac{C_{FP}}{B_{TP}}$, measuring, in essence, to what extent the total benefit by all true positives outweighs the total cost of all false positives [23, 37]. The decision curve for ADNEX is plotted in Additional file 3. Other utility-respecting evaluations of predictive performance conditional on the threshold have also been proposed [38, 39]. Rather than summarizing the clinical utility of a model at a range of relevant risk thresholds, the partial AUC summarizes diagnostic accuracy over a clinically interesting range of specificity (or sensitivity) [40]. The partial AUC has some limitations, such as conditioning on a classification result that varies from one sample to the next, and not taking the cost–benefit ratio of a false positive versus a true positive into account.

To compare models, for example, a model with and without a novel biomarker, one may be tempted to believe its clinical utility is demonstrated if patients with the disease move to a higher risk group and patients without the disease move to a lower risk group after addition of the marker to a model; this is measured by reclassification statistics such as net reclassification improvement. The results of such an analysis again depend on the chosen risk thresholds to define groups; in addition, they may favor miscalibrated models [41–43]. When comparing risk models based on partial AUC, one could be comparing models at different ranges of risk thresholds. Better alternatives are to calculate the difference in (full) AUC, use likelihood-based statistics, or to conduct a decision curve analysis to compare competing models when accounting for costs and benefits of decisions [23, 37].

## Conclusion

Clinical prediction models are helpful for decision-making in clinical practice. For this purpose, reliable continuous risk estimates are key. If risk thresholds are needed to identify high-risk patients, optimal thresholds cannot be calculated from the data on predictors and the true disease status alone. Instead, the choice of threshold should reflect the harms of false positives and the benefits of true positives, which varies depending on the clinical context. We propose focusing on methods that evaluate predictive performance independent of risk thresholds (such as AUC and calibration plots) or incorporate a range of risk thresholds (such as decision curve analysis). If a risk threshold is required, we advise the performance of a health economic analysis after the model has been validated.

## Supplementary information

**Additional file 1.** Csv file containing the predicted probabilities of malignancy by the ADNEX model and the true outcomes (1 = malignant, 0 = benign).

**Additional file 2.** Word file containing annotated R code to replicate the analyses.

**Additional file 3.** Decision curve analysis comparing the utility of the ADNEX model for clinical decision-making to treating all patients and treating none of the patients.

## Data Availability

The dataset supporting the conclusions of this article is included within the article and its additional files.
