# Supplementary material for: Three myths about risk thresholds for prediction models
Source: BMC Med. 2019 Oct 25;17:192. doi: 10.1186/s12916-019-1425-3 (PMC6814132; doi:10.1186/s12916-019-1425-3)
Supplement: Supplementary file 2 — Additional file 2. Word file containing annotated R code to replicate the analyses. [file 12916_2019_1425_MOESM2_ESM.docx]

Three myths about risk thresholds in prediction models

**Laure Wynants, July 12th 2019**

This document shows the R code and output needed to replicate the analysis in the paper “Three myths about risk thresholds in prediction models” by Wynants et al.

Boxes with a gray background contain R code and output. Code can be copy-pasted into R. Text preceded by # are comments not interpreted by R. Black text preceded by ## is R output, brown text preceded by # are comments to explain specific parts of the code to you.

In between the shaded boxes is text like this, with annotations to the R code and output.

# Set up the libraries and data needed for the analysis

library(tidyverse)

## -- Attaching packages ---------------------------------------------------------- tidyverse 1.2.1 --

## v ggplot2 3.1.0 v purrr 0.2.5
## v tibble 2.0.1 v dplyr 0.7.8
## v tidyr 0.8.2 v stringr 1.3.1
## v readr 1.3.1 v forcats 0.3.0

## -- Conflicts ------------------------------------------------------------- tidyverse_conflicts() --
## x dplyr::filter() masks stats::filter()
## x dplyr::lag() masks stats::lag()

library(OptimalCutpoints)
library(epiR)

## Loading required package: survival

## Package epiR 0.9-99 is loaded

## Type help(epi.about) for summary information

##

mypath <- "C:\\Users\\u0060918\\Documents\\methods\\common misconceptions on risk thresholds\\code\\data.csv"
data <- as_tibble(read_csv(file = mypath)) #specify the correct path for the data

## Parsed with column specification:
## cols(
## pred = col_double(),
## outcome = col_double()
## )

# Take a look at the data

glimpse(data)

## Observations: 2,403
## Variables: 2
## $ pred <dbl> 0.040514624, 0.035508552, 0.159263818, 0.713899504, 0....
## $ outcome <dbl> 0, 0, 1, 1, 0, 0, 1, 0, 0, 0, 1, 1, 0, 0, 0, 0, 0, 0, ...

The data contains 2403 observations, each with a predicted risk (pred) and the true outcome.

# A. Prevalence as a threshold

t_prev <- mean(data$outcome)
round(t_prev*100)

## [1] 41

41% of patients in the database experience the event. Patients with a risk below the event fraction could be considered low risk, and patients with a higher risk could be considered high risk.

# B. A threshold that minimizes misclassification

misclass <-
 summary(
 optimal.cutpoints(
 X = 'pred',
 status = 'outcome',
 tag.healthy = 0,
 data = as.data.frame(data),
 methods = c("MCT") #MCT minimizes Misclassification Cost Term, assuming equal costs for a false positive decision and a false negative decision. This gives you the threshold that minimizes misclassification error.
 )
 )
t_misclass <- misclass$MCT$Global$optimal.cutoff$cutoff[1]
round(t_misclass*100)

## [1] 31

As the object misclass shows, there are four different thresholds that yield the same total number of misclassified (false positive or false negative) patients: 30.7%, 30.9%, 31.2% and 33%. As the choice is arbirtary, we will proceed with the first of these.

# C. A utility-based threshold using the costs of each classification

CFP <- 5 #cost of a false positive
CTP <- 15 #cost of a true positive
CFN <- 95 #cost of a false negative
CTN <- 0 #cost of a true negative

t_utility <- (CFP - CTN) / (CFP + CFN - CTP - CTN)
round(t_utility * 100)

## [1] 6

The optimal cost-based threshold (assuming a calibrated risk model) is 6%.

# Figure 1. Frequencies of predicted risks of malignancy, and three possible risk thresholds. The colored area of each bin is proportional to the numbers of patients with benign and malignant tumors.

data$tumor <- factor(ifelse(data$outcome == 0, "benign", "malignant"))

p <- ggplot(data = data, mapping = aes(x = pred, fill = tumor, color = tumor))

p_out <-
 p + geom_histogram(bins = 100, alpha = 0.9) +
 labs(x = "predicted risk of malignany", y = "frequency count") +
 scale_fill_brewer(palette="Set2") +
 scale_color_brewer(palette="Set2") +
 scale_x_continuous(labels=scales::percent) +
 theme_bw() +
 theme(
 legend.position = "bottom",
 panel.border = element_blank(),
 panel.grid.major = element_blank(),
 panel.grid.minor = element_blank(),
 axis.line = element_line(colour = "black")
 ) +
 geom_vline(xintercept = c(t_prev, t_misclass, t_utility), color = "steelblue") +
 geom_text(
 aes(x = t_prev, label = "prevalence as threshold", y = 205),
 colour = "steelblue",
 angle = 90,
 vjust = 1.2
 ) +
 geom_text(
 aes(x = t_misclass, label = "threshold minimizing misclassification", y = 157),
 colour = "steelblue",
 angle = 90,
 vjust = 1.2
 ) +
 geom_text(
 aes(x = t_utility, label = "cost-based threshold", y = 210),
 colour = "steelblue",
 angle = 90,
 vjust = 1.2
 )

p_out


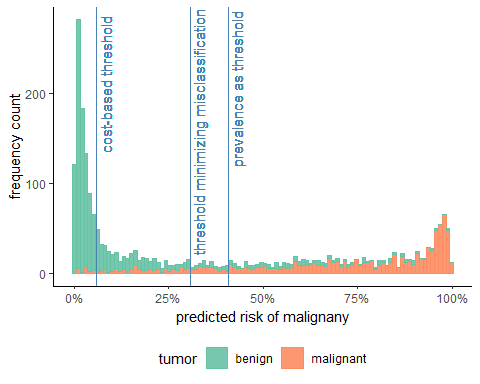


ggsave("histogram.pdf", plot = p_out, height = 10, width = 15, units = "cm")

# Table 2. Classification statistics for a selection of thresholds (95% confidence interval)

#Repeat to calculate the sens, spec, PPV and NPV for a number of reasonable thresholds,
#including 0.001, 0.999, t_prev, t_misclass and t_utility

t <- t_utility
crosstab <- table(test_negative = (data$pred) < t, benign = (data$outcome == 0))
epi.tests(crosstab)

## Outcome + Outcome - Total
## Test + 958 548 1506
## Test - 22 875 897
## Total 980 1423 2403
##
## Point estimates and 95 % CIs:
## ---------------------------------------------------------
## Apparent prevalence 0.63 (0.61, 0.65)
## True prevalence 0.41 (0.39, 0.43)
## Sensitivity 0.98 (0.97, 0.99)
## Specificity 0.61 (0.59, 0.64)
## Positive predictive value 0.64 (0.61, 0.66)
## Negative predictive value 0.98 (0.96, 0.98)
## Positive likelihood ratio 2.54 (2.38, 2.71)
## Negative likelihood ratio 0.04 (0.02, 0.06)
## ---------------------------------------------------------

At the utility-based threshold with the specified costs, the sensitivity is 98% and the specificity 61%. The PPV is 64% and the NPV is 98%.

# Appendix Decision Curve Analysis

#Download the R-function from www.decisioncurveanalysis.org

mypath_dca <- "C:\\Users\\u0060918\\Documents\\methods\\common misconceptions on risk thresholds\\code\\dca.r"
source(mypath_dca)

data$ADNEX <- data$pred
dca(data=as.data.frame(data), outcome="outcome", predictors="ADNEX", xstop=0.5)


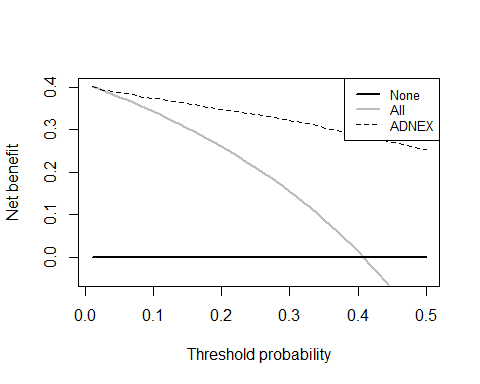


## $N
## [1] 2403
##
## $predictors
## predictor harm.applied probability
## 1 ADNEX 0 TRUE
##
## $interventions.avoided.per
## [1] 100
##
## $net.benefit
## threshold all none ADNEX
## 1 0.01 0.401841974 0 0.4017537
## 2 0.02 0.395738320 0 0.3969613
## 3 0.03 0.389508818 0 0.3928037
## 4 0.04 0.383149535 0 0.3904148
## 5 0.05 0.376656373 0 0.3871476
## 6 0.06 0.370025057 0 0.3842449
## 7 0.07 0.363251133 0 0.3823715
## 8 0.08 0.356329950 0 0.3788742
## 9 0.09 0.349256653 0 0.3766126
## 10 0.10 0.342026171 0 0.3744394
## 11 0.11 0.334633207 0 0.3726942
## 12 0.12 0.327072220 0 0.3690841
## 13 0.13 0.319337418 0 0.3668642
## 14 0.14 0.311422737 0 0.3637604
## 15 0.15 0.303321828 0 0.3617047
## 16 0.16 0.295028040 0 0.3582427
## 17 0.17 0.286534402 0 0.3541507
## 18 0.18 0.277833602 0 0.3512175
## 19 0.19 0.268917968 0 0.3493113
## 20 0.20 0.259779442 0 0.3473783
## 21 0.21 0.250409562 0 0.3449170
## 22 0.22 0.240799428 0 0.3434916
## 23 0.23 0.230939680 0 0.3396350
## 24 0.24 0.220820466 0 0.3377138
## 25 0.25 0.210431405 0 0.3354141
## 26 0.26 0.199761559 0 0.3324898
## 27 0.27 0.188799389 0 0.3301467
## 28 0.28 0.177532714 0 0.3284089
## 29 0.29 0.165948667 0 0.3238792
## 30 0.30 0.154033648 0 0.3211462
## 31 0.31 0.141773267 0 0.3186235
## 32 0.32 0.129152285 0 0.3150718
## 33 0.33 0.116154558 0 0.3133645
## 34 0.34 0.102762960 0 0.3089825
## 35 0.35 0.088959314 0 0.3046512
## 36 0.36 0.074724303 0 0.3006918
## 37 0.37 0.060037387 0 0.2972739
## 38 0.38 0.044876700 0 0.2944975
## 39 0.39 0.029218941 0 0.2922167
## 40 0.40 0.013039256 0 0.2892218
## 41 0.41 -0.003688892 0 0.2879522
## 42 0.42 -0.020993873 0 0.2825491
## 43 0.43 -0.038906046 0 0.2795701
## 44 0.44 -0.057457939 0 0.2754295
## 45 0.45 -0.076684447 0 0.2707979
## 46 0.46 -0.096623048 0 0.2673818
## 47 0.47 -0.117314049 0 0.2637819
## 48 0.48 -0.138800858 0 0.2609879
## 49 0.49 -0.161130286 0 0.2565829
## 50 0.50 -0.184352892 0 0.2542655
##
## $interventions.avoided
## threshold ADNEX
## 1 0.01 -0.8739076
## 2 0.02 5.9925094
## 3 0.03 10.6533500
## 4 0.04 17.4365377
## 5 0.05 19.9334166
## 6 0.06 22.2777084
## 7 0.07 25.4027703
## 8 0.08 25.9259259
## 9 0.09 27.6598696
## 10 0.10 29.1718685
## 11 0.11 30.7948398
## 12 0.12 30.8087113
## 13 0.13 31.8063959
## 14 0.14 32.1502883
## 15 0.15 33.0836454
## 16 0.16 33.1876821
## 17 0.17 33.0126557
## 18 0.18 33.4304342
## 19 0.19 34.2729483
## 20 0.20 35.0395339
## 21 0.21 35.5527812
## 22 0.22 36.4090342
## 23 0.23 36.3892960
## 24 0.24 37.0162297
## 25 0.25 37.4947982
## 26 0.26 37.7764973
## 27 0.27 38.2161187
## 28 0.28 38.7967422
## 29 0.29 38.6657483
## 30 0.30 38.9929255
## 31 0.31 39.3634301
## 32 0.32 39.5079068
## 33 0.33 40.0395970
## 34 0.34 40.0308438
## 35 0.35 40.0570715
## 36 0.36 40.1720072
## 37 0.37 40.3943269
## 38 0.38 40.7275993
## 39 0.39 41.1355464
## 40 0.40 41.4273824
## 41 0.41 41.9678654
## 42 0.42 41.9178408
## 43 0.43 42.2166091
## 44 0.44 42.3674952
## 45 0.45 42.4700606
## 46 0.46 42.7310065
## 47 0.47 42.9746505
## 48 0.48 43.3104453
## 49 0.49 43.4762669
## 50 0.50 43.8618394
