# Supplementary material for: Three myths about risk thresholds for prediction models
Source: BMC Med. 2019 Oct 25;17:192. doi: 10.1186/s12916-019-1425-3 (PMC6814132; doi:10.1186/s12916-019-1425-3)
Supplement: Supplementary file 3 — Additional file 3. Decision curve analysis comparing the utility of the ADNEX model for clinical decision-making to treating all patients and treating none of the patients. [file 12916_2019_1425_MOESM3_ESM.docx]

**Additional file 3. Decision curve analysis comparing the utility of the ADNEX model for clinical decision-making to treating all patients and treating none of the patients.**


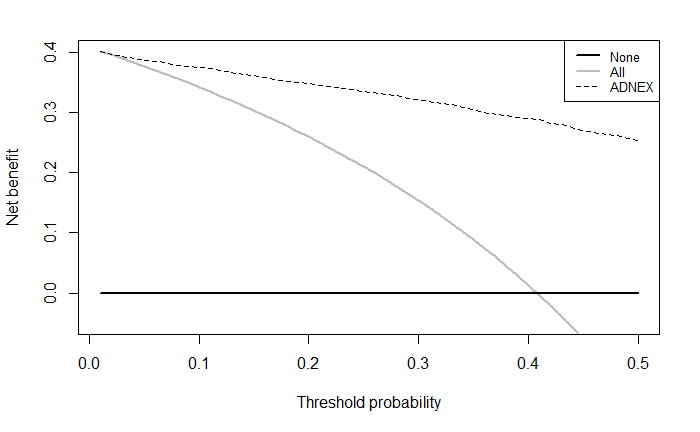


The net benefit (the vertical axis in the plot) balances the benefits of true positives against the harms of false positives on a single scale, by using a weighting factor w for false positives:

net benefit = (number of true positives – w × number of false positives) / N

The weighting factor is the risk threshold odds (given on the horizontal axis), which equals C_FP_/B_TP_, the cost-benefit ratio of a false positive versus a true positive. The higher the risk threshold, the larger the weight, and the larger the penalization for false positives.

The net benefit can be used to compare models or markers. A model should minimally be compared to treating all and treating none of the patients. If the net benefit of the model is lower than that of treat all or treat none, it has no clinical utility for decision-making, and better decisions would be obtained without the model. For an in-depth discussion of decision curve analysis, we refer the reader elsewhere [23, 37].
